# A little bit of sex matters for genome evolution in asexual plants

**DOI:** 10.3389/fpls.2015.00082

**Published:** 2015-02-20

**Authors:** Diego Hojsgaard, Elvira Hörandl

**Affiliations:** Department of Systematics, Biodiversity and Evolution of Plants, Albrecht-von-Haller Institute for Plant Sciences, Georg-August University of Göttingen, Göttingen, Germany

**Keywords:** apomixis, Muller’s ratchet, Meselson effect, polyploidy, heterozygosity

## Abstract

Genome evolution in asexual organisms is theoretically expected to be shaped by various factors: first, hybrid origin, and polyploidy confer a genomic constitution of highly heterozygous genotypes with multiple copies of genes; second, asexuality confers a lack of recombination and variation in populations, which reduces the efficiency of selection against deleterious mutations; hence, the accumulation of mutations and a gradual increase in mutational load (Muller’s ratchet) would lead to rapid extinction of asexual lineages; third, allelic sequence divergence is expected to result in rapid divergence of lineages (Meselson effect). Recent transcriptome studies on the asexual polyploid complex *Ranunculus auricomus* using single-nucleotide polymorphisms confirmed neutral allelic sequence divergence within a short time frame, but rejected a hypothesis of a genome-wide accumulation of mutations in asexuals compared to sexuals, except for a few genes related to reproductive development. We discuss a general model that the observed incidence of facultative sexuality in plants may unmask deleterious mutations with partial dominance and expose them efficiently to purging selection. A little bit of sex may help to avoid genomic decay and extinction.

## INTRODUCTION

**FIGURE 1:**
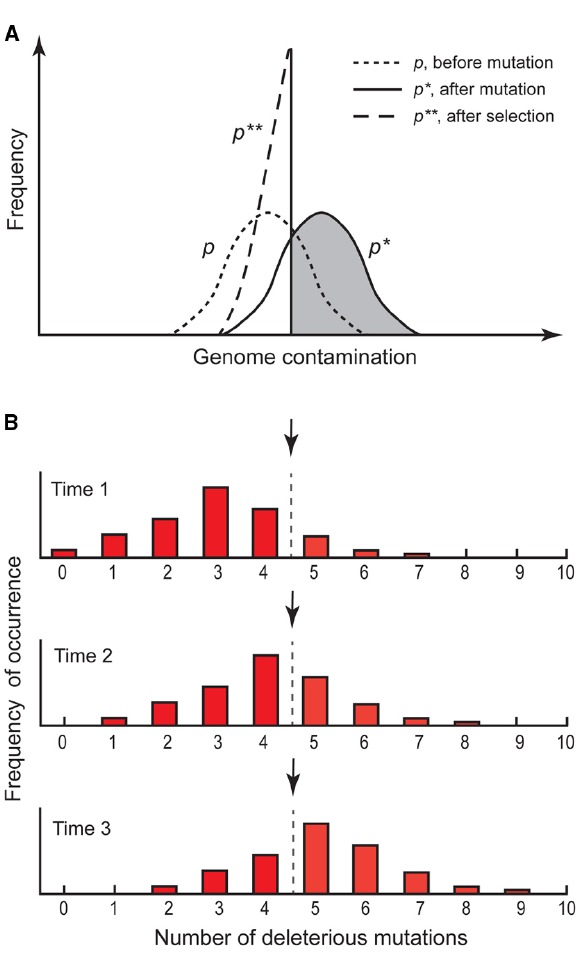
**Principles of Muller’s ratchet. (A)** Scheme of distributions (*p*) of mutations in a sexual population. Before mutation, distribution in the population is (*p*), after mutation, distribution shifts upward to p*. After recombination and selection against mutants, individuals in the gray part remain sterile and die, and the distribution goes backward to p**. At equilibrium the means of *p* and p** are equal (redrawn after Kondrashov, 1988). **(B)** Scheme of mutational load distributions in an asexual population. Initially, genotypes with zero mutations exist in the population, but are lost over time by drift. Without recombination, the class with zero or few mutations cannot be restored, and consequently mutations accumulate until a threshold level of extinction (arrow) is reached (redrawn after Maynard Smith, 1988).

Currently, the understanding of evolution patterns of genomes on different phylogenetic groups is a hot topic in evolutionary biology. The arrival of next generation sequencing (NGS) technologies and the generation of huge amounts of genomic data is allowing researchers to dig in the past and better resolve organisms’ natural history as well as evolutionary enigmas. One of such enigmas is the predominance of sex in nature (Otto, 2009). One of the most prominent theories explaining the benefits of sex (for broad analyses see, e.g., Bell, 1982; Birdsell and Wills, 2003) proposes that sexuality protects the genome from the accumulation of deleterious mutations (Muller, 1964; Kondrashov, 1988; Hörandl, 2009; Figure 1). Here we will discuss theoretical assumptions and empirical possibilities of presence/absence of meiosis for asexual plant genome evolution in the light of unexpected recent findings on sexual/asexual taxa of *Ranunculus*.

Sexuality is a crucial factor molding the genomic features of eukaryotes. In plants, the formation of a new individual through sexuality involves an alternation between the sporophytic (2n) and the gametophytic (n) generations via meiosis and gamete fusion, the two mechanisms that create new genetic combinations. Additionally, outcrossing further potentiates genetic variation in populations. Thus, with few exceptions, every single sexual organism has a distinctive genotype that differentiates it from parents and siblings. Therefore, meiosis is the main source of genetic recombination and mixis, and segregates genetic factors in the offspring creating genetic variation. By doing so, meiosis and sexuality allows natural selection purging a lineage from harmful mutations. Because plant meiosis produces spores (mega- and microspores) and these spores develop into female and male gametophytes, in which considerable percentages of genes are being expressed (Joseph and Kirkpatrick, 2004), selection in a sexual plant will act at two developmental stages: during gametophyte development (haploid gametophytic selection) and after the formation of the zygote (sporophytic selection) Hörandl (2013).

In contrast, by circumventing or suppressing meiosis and syngamy, asexual organisms skip the alternation of generation cycle and hence elude the ploidy-phase change step. In angiosperms, asexually-derived individuals can be formed either as consequence of vegetative propagation, or of asexual seed formation (apomixis), a trait that is taxonomically widespread in plants (Hojsgaard et al., 2014a). While the first involves extra vegetative growth and fragmentation without undergoing the single-cell stage and embryogenesis, the latter comprises the development of a new organism out of an unreduced, unfertilized egg cell, embryogenesis and seed formation (Mogie, 1992). In apomictic plants, a combination of complex developmental features avoid recombination and reductional steps present in the normal sexual reproductive process, thus developing a seed carrying a clonal embryo (Asker and Jerling, 1992).

A central fact for genome evolution, however, is that apomixis in angiosperms is rarely obligate. Apomictic plants produce asexual and sexual progeny within the same offspring generation, i.e., from different ovules and seeds in the same mother plant, and therefore asexuality is facultative. Consequently, a proportion of the offspring represents recombinants, but frequencies of sexuality vary a lot among genera, species and different modes of apomixis (e.g., Aliyu et al., 2010; Sartor et al., 2011; Šarhanová et al., 2012; Noyes and Givens, 2013; Hojsgaard et al., 2013, 2014a). The role that facultative sexuality and genetically highly diverse apomictic populations play in the evolution of angiosperms is still unclear.

## THEORETICAL SIGNIFICANCE OF APOMIXIS FOR THE EVOLUTION OF THE PLANT GENOME

**FIGURE 2:**
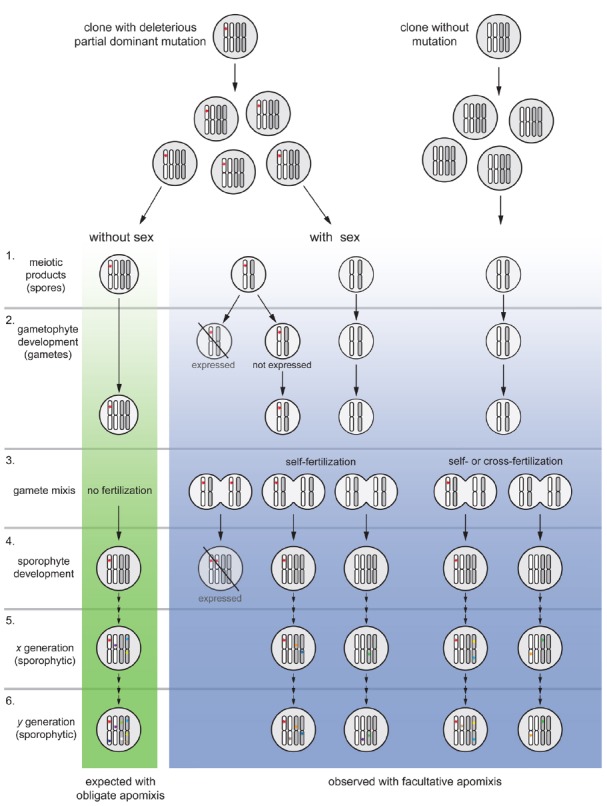
**Model of purging mutations in a tetraploid, facultative apomictic plant lineage (blue column) compared to an obligate apomict (without meiosis; green column).** For simplicity, the model is presented for a new self-fertile allotetraploid lineage with regularly reduced male gametes; and partial dominant mutations are considered to be deleterious and expressed a 50% penetrance. Moreover, preferred homolog pairing is assumed during meiosis I (e.g., Comai, 2005) and considers only the perspective of a mutated deleterious allele, all other alleles pondered to be functionally equivalent. The effects of absence or presence of meiosis on mutation accumulation are illustrated after one generation following the occurrence of mutation (stages 1–4), and after several generations of obligate (without sex) or facultative apomixis (with residual levels of sex; stages 5–6). 1. Once a deleterious mutation (red star) with a 50% penetrance is loaded onto the clonal offspring, without sex only unreduced female gametes rise (clonal) progeny. With sex recombinant spores are formed. 2. Expression of mutated alleles and deleterious effects would appear only in those gametophytes with a ploidy-phase change; thus, 50% of haploid gametes would be eliminated, biasing expected progeny proportions (but not progeny types). 3. During gamete mixis, parthenogenetic embryo development avoids egg-cell fertilization in apomictic female gametophytes while meiotic ones can produce an array of progeny types upon self- or cross-fertilization syndromes. 4. A dosage increase (to duplex condition) and full expression of deleterious effects is expected in some recombinant offspring during sporophyte development, but not in non-recombinant ones. Only individuals carrying a low allele dosage (simplex condition) will remain in the population together with those without the mutation. 5–6. After a number of generations, mutations will gradually appear and added up to the genetic load in the obligate apomictic lineage. In the facultative apomictic lineage, occasional sex will segregate mutated alleles and purging selection will eliminate gametophytes and sporophytes with certain allelic dosages (as in stages 1–4). On the long run, an obligate apomictic genotype (left) will become sooner extinct compared to a facultative apomictic lineage which is continuously purged. The model does not yet consider possible purging effects via conversion during meiosis, and does not quantify facultative sexuality and actual frequencies of spore formation. The model fits to higher ploidy levels if the same penetrance level is assumed in mutated alleles. Assorted colored mutations represent independent events arisen randomly in the genome at different times.

Sexuality has the effect that deleterious mutations appear in various genotypic configurations in the offspring. Thus, harmful mutations expressed under different states (e.g., homozygous, dominant heterozygous, etc.) will negatively affect the genotype’s fitness and natural selection will remove such genotypes and purge the lineage from an increase in the mutational load. Evolutionary benefits of purging a lineage from mutation accumulation have long been seen as a major advantage of sexuality (Muller, 1964; Kondrashov, 1988; Figure 1A). Apomictic plants, by circumventing genetic reshuffling mechanisms, inherit the same genomic features of their female parental genome. Without recombination, once a genotype acquire a spontaneous mutation cannot reconstitute a non-mutated genotypic state (Figure 1B and 2). Thus, a deleterious mutation in any asexual individual will be transmitted to all the offspring and loaded onto the gene pool of that clonal lineage. In any segment of a genome with absence of recombination, the number of random independent mutations is expected to increase because a mutational load smaller than the least-loaded lineage can never be generated (the ratchet mechanism, Muller, 1964; the hatchet mechanism, Kondrashov, 1988). Over time, drift will ultimately lead to a loss of genotypes with a lower mutational load and, once a threshold on mutational load is reached, to extinction of the asexual lineage (Figure 1B; Kimura et al., 1963; Muller, 1964; Felsenstein, 1974). Moreover, genetic interference effects between loci (Hill-Robertson effect, Hill and Robertson, 1966) may increase effects of mutations (Kondrashov, 1988). The genetic load of clonal lineages will reduce their fitness and obstruct further adaptation, driving those lineages to an early extinction (Maynard Smith, 1978; Bell, 1982).

Apomixis in plants is nearly exclusive associated polyploids, and often it is a result of hybridity (e.g., Koch et al., 2003; Paun et al., 2006a; Pellino et al., 2013). Polyploidy can accelerate mutation accumulation since additional gene copies represent additional mutational sites. Effects of deleterious mutations will reduce the mean fitness of any individual (and ultimately of the population) with a rate c U, where c stand for the ploidy level and U for the mutation rate per haploid genome (Gerstein and Otto, 2009). However, the effects of single deleterious recessive mutations in heterozygous states can be masked by a functional gene copy of the wild or dominant allele in a diploid organism (Crow and Kimura, 1970; Kondrashov and Crow, 1991; Otto and Whitton, 2000). After a prolonged diploid stage, the return to haploidy leads to the expression of accumulated, but previously masked deleterious recessive alleles, and selection against mutations (Crow and Kimura, 1970; Hörandl, 2009). However, in a polyploid plant with more than two allele copies per locus, accumulated recessive mutations in heterozygous states may still be masked after a return to haploidy. Thus, masking effects will be stronger and recessive mutations may not be effective unless they show certain level of dominance (i.e., partial dominance). So far few genomic studies are available to understand the forces underlying mutation dynamics in polyploid plants. In theory, absence of haploid gametophytic selection plus masking of recessive alleles in polyploid condition should increase the mutational load compared to sexuals.

Allelic sequence divergence, the so-called Meselson effect, is another consequence of long-term asexuality (Mark Welch and Meselson, 2000). Asexual seed reproduction, by suppressing meiosis promotes the divergence between allelic sequences by neutral mutations (Kimura and Crow, 1964). Due to the loss of sexuality, alleles within lineages will gain neutral differences at a much higher rate than the normal substitution rates observed between alleles in sexual populations (Birky, 1996). Until now, the presence of genome-wide allele sequence divergence significantly larger than those in inter-lineages have been hard to prove. Processes such as gene conversion, mitotic recombination, efficient DNA repair, meiotic parthenogenesis in animals (automixis), occasional sex, ploidy reduction, and hybridization can moderate or remove sequence divergence (e.g., Schön and Martens, 1998; Schaefer et al., 2006; Liu et al., 2007; Mark Welch et al., 2008; Flot et al., 2013). Effects of facultative sexuality on allelic sequence divergence in plants remain unknown.

## IS A LITTLE BIT OF SEX SUFFICIENT TO AVOID MUTATION ACCUMULATION?

A recent transcriptome study of the *Ranunculus auricomus* complex, a system of diploid sexual species and hexaploid apomictic hybrids, in fact showed Meselson-like sequence divergence effects, but data did not support the idea of mutation accumulation (Pellino et al., 2013). Analyses of Muller’s ratchet on high-quality single nucleotide polymorphisms (SNPs) and indels obtained from RNA-seq revealed for 1231 annotated genes that ratios of non-synonymous vs. synonymous substitutions (dN/dS) were mostly clearly below one, and did not differ significantly between apomictic-apomictic, apomictic-sexual and sexual-sexual comparisons (Pellino et al., 2013). A number of all annotated genes showed high dN/dS ratios (outlier values; see Figure 3 in Pellino et al., 2013), and hence appeared to be under divergent selection. A gene ontology analysis of outliers showed that a small proportion of those genes (*n* = 62; 6.7%; Table 5 in Pellino et al., 2013) were associated with processes involved in meiosis and gametogenesis. Strikingly, most such outlier genes (*n* = 41; 66.1%) were found in the apomictic-sexual comparison and thus indicated a significant enrichment of genes associated to reproductive shifts during ovule development when compared to those outliers in sexual (or apomictic) genomes. Whether these mutations have positive or negative effects, needs further investigations. However, most mutations that are under selection do have strongly deleterious effects (e.g., Loewe and Hill, 2010). Since we analyzed only RNA sequences we assume that the observed non-synonymous substitutions mostly have negative effects (dominant or partial dominant). If deleterious mutations would have accumulated genome-wide in the apomicts–a situation expected following the nature of the genetic code and transition/transversion rates (e.g., Yang and Bielawski, 2000)—they would drive the dN/dS ratios over 1. One interpretation of such results is that apomicts in *Ranunculus* are evolutionarily too young (c. 70,000 years) to have accumulated significant mutations and hence dN/dS ratios are still similar to those in sexual putative parentals.

The alternative explanation, however, assumes that apomicts do not accumulate genome-wide deleterious mutations because facultative sexuality purges deleterious mutations (Figure 2). Detailed developmental studies revealed that apomictic hexaploid *Ranunculus* hybrids show varying proportions of sexually formed seed in all genotypes, with a grand mean of 29.1% (Hojsgaard et al., 2014b). Reduced seed set and lower pollen quality of apomicts compared to sexuals (Hörandl, 2008; Hojsgaard et al., 2014b) indicate negative effects of apomixis on fitness parameters. Population genetic studies data indicate considerable genetic diversity within and among populations (Paun et al., 2006a). Considering the expected turn-over of recombinant individuals in natural populations, regular sexuality in facultative apomicts can purge mutations via two mechanisms: First, ploidy reduction in a diploid plant can already unmask recessive deleterious mutations in the gametophyte and expose it to purging selection (Hörandl, 2009), while in a polyploid mutations showing partial dominance would be exposed to selection during haploidy (see Figure 2, stages 1 and 2); since the gametophytes represent few-celled mini-organisms, a high proportion of the genome is expressed and exposed to selection at this stage. In fact, proportions of sexual development decrease during haploid gametophyte development (Hojsgaard et al., 2013, 2014a). Second, via recombination, mutations will segregate and offspring with variable mutational load will be formed. Additionally, self-fertilization will generate zygotes with higher doses (e.g., hemizygous) of partially dominant mutated alleles, and consequently mutations will become “unmasked” and fully exposed to purging selection in the offspring (see Figure 2, stage 3). Thus, those genotypes where deleterious mutations are being expressed will be eliminated upon dosage level and only individuals carrying mutated alleles at low dosages will persist in the population (see Figure 2, stage 4). The lineage will consequently be regularly purged by eliminating genotypes carrying these mutations. Hence, novel mutations in a facultative will add up slower than in obligate apomicts in which each mutation is added to the mutational load (Figure 2, stages 5 and 6).

The ultimate efficacy will certainly depend on the level of functional meiosis and sexuality occurring in the population. Besides this, purging is expected to be even more efficient under diverse conditions. For example, depending on the level of penetrance and dominance of the mutation, purging would be faster as phenotypic effects would become exposed to natural selection at different dosages. A higher purging efficacy is expected with inbreeding (e.g., Agrawal and Chasnov, 2001). In fact, in a clonal population established from an apomictic mother, all neighboring individuals will carry the same deleterious mutation in their gametes. Occasional facultative sex among individuals will occur among the same genotypes, which is possible because of self-compatibility of apomictic plants (including hexaploid *Ranunculus*; Hörandl, 2008, 2010). Hence, plants effectively conduct self-fertilization, even if cross-pollination takes place among individuals (clone-mates); thus, alleles carrying deleterious mutations may rapidly increase in their dosage, and consequently their effects will be exposed to selection (Figure 2).

Another mechanism to increase efficacy of selection can be assumed from epistasis. If additional (recessive or non-recessive) deleterious mutations lead to a larger decrease of fitness because of negative interactions of these genes, then even truncating selection can act and rapidly eliminate this genotype (Kondrashov, 1988). Hence, despite nearly-obligate apomictic clones within a population could accumulate mutations for some generations, fitter recombinant genotypes with a lower mutational load will continuously replace them (see Figure 2). Population genetic data on *Ranunculus* strongly support this hypothesis of clonal turnover (Paun et al., 2006b). Consequently, Muller’s ratchet is halted or at least slowed down for the lineage as whole. A fourth possibility to avoid mutation accumulation is a large effective population size (Kondrashov, 1988) and/or high migration rates amid populations (Whitton et al., 2008). Hence, within populations genetic variability will be kept at high levels and selection will act on different genotypes. Large geographical distribution and clonal diversity of the hexaploid hybrids (see Paun et al., 2006a,b) suggest that also this factor contribute to genome evolution in *Ranuncu lus*.

The evolutionary consequences of facultative apomixis in plants have so far received little attention. Mendelian genetic studies on control mechanism of apomixis in angiosperms suggest that the apomixis-controlling genomic regions occur -in general- in a heterozygous state (Ozias-Akins and van Dijk, 2007). In *Ranunculus auricomus*, quantitative expression of apomixis is dosage-dependent on the apospory factor (A), which is a dominant Mendelian factor with variable penetrance in the sporophyte, but with lethal effects in haploid or homozygous states (Nogler, 1984). Consequently, (A) appears always heterozygous with the wild type allele a in various allelic configurations, which means that apomixis cannot become completely obligate (Nogler, 1984). The long term effects of facultative sexuality remain to be studied. Overall, data (Pellino et al., 2013) suggest that apomictic polyploid lineages on the one hand accumulate Meselson-effect-like neutral substitutions in divergent gene copies, and on the other hand, mask partially dominant deleterious alleles in clones, which may become exposed to purifying selection via facultative sexuality. Comprehensive empirical studies will be needed to further test theoretical models and answer the question of how much and to what extend “a little bit of sex” protects apomictic plants from genomic decay and extinction.

### Conflict of Interest Statement

The authors declare that the research was conducted in the absence of any commercial or financial relationships that could be construed as a potential conflict of interest.
